# Isolation and characterization of antioxidant peptides from *oyster* (*Crassostrea rivularis*) protein enzymatic hydrolysates

**DOI:** 10.1002/fsn3.3058

**Published:** 2022-09-25

**Authors:** Hui Huang, Jianwei Cen, Daqiao Yang, Laihao Li, Chunsheng Li, Xianqing Yang, Yueqi Wang, Xiao Hu, Jinxu Wang, Qiuxing Cai

**Affiliations:** ^1^ Key Laboratory of Aquatic Product Processing, Ministry of Agriculture and Rural Affairs, Guangdong Provincial Key Laboratory of Fishery Ecology Environment South China Sea Fisheries Research Institute, Chinese Academy of Fishery Science Guangzhou China; ^2^ College of Food Science and Engineering Ocean University of China Qingdao China; ^3^ School of Life Sciences and Food Technology Hanshan Normal University Chaozhou China; ^4^ Guangxi Colleges and Universities Key Laboratory Development and High‐value Utilization of Beibu Gulf Seafood Resources, College of Food Engineering Beibu Gulf University Qinzhou China

**Keywords:** antioxidant activity, *Crassostrea rivularis*, enzymatic hydrolysis, oxidative damage, peptide

## Abstract

Peptides from oysters have several bioactive functions. In this study, we identified antioxidant peptides from oysters (*Crassostrea rivularis*) and investigated their structure–function relationship. We used an 8 kDa molecular‐weight (MW) cut‐off membrane and semiprep reversed‐phase liquid chromatography to collect five peptides (F1–F5) and identified the highest‐abundance ion‐peak sequences AWVDY (F1), MSFRFY(F2), EPLRY(F3), RKPPWPP(F4), and YAKRCFR(F5) having MWs of 652, 850, 676, 877, and 943 Da, respectively, using ultra‐performance liquid chromatography‐quadrupole/time‐of‐flight tandem mass spectrometry. These peptides exhibited high antioxidant activities, similar to butylated hydroxytoluene, reduced glutathione, and ascorbic acid. F5 demonstrated the highest scavenging activity for DPPH radicals (IC_50_ = 21.75 μg/ml), hydroxyl radicals (IC_50_ = 18.75 μg/ml), and superoxide radicals (IC_50_ = 11.00 μg/ml), while F3 demonstrated the highest reducing power. Furthermore, F5 significantly protected Caco‐2 cells from H_2_O_2_‐induced oxidative damage. These results suggest that the antioxidant peptide F5 is a promising food additive that protects against oxidative damage.

## Graphical abstract

Five obtained peptides exhibited high radical scavenging activities in the following order: F5 > F3 > F1 > F2 > F4.; F5 has the highest activities for DPPH‐IC50 was 21.75 μg/mL, hydroxyl‐IC50 was 18.75 μg/mL, and Superoxide‐IC50 was 11.00 μg/mL and the reducing power was 2.54 OD700.; F5 had the highest antioxidant activity and not only prevented the cell injury but also promoted the cell growth.

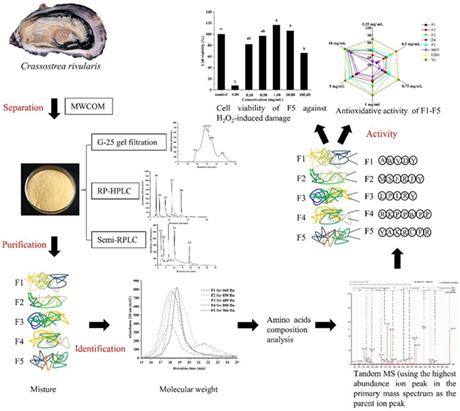

## 1 INTRODUCTION

Excessive free radicals and reactive oxygen species (ROS) promote damaging reactions in many cellular components, thereby creating oxidative stress, and promoting the onset of diseases such as cancer, gastric ulcers, arthritis, premature aging, inflammation, and atherosclerosis (Pan et al., 2017; Shan et al., 2015; Suthisamphat et al., 2020). In food products, the use of synthetic antioxidants such as butylated hydroxyanisole and butylated hydroxytoluene (BHT) is under strict regulation because of their potential health hazards and toxic effects (Mirzaei et al., 2015). Therefore, there is a great need for alternative antioxidants that are safe and exhibit high activity.

Recently, an increasing number of studies have focused on antioxidant peptides, which possess many advantages including high activity, innocuity, and easy absorption (Tadesse & Emire, 2020). Antioxidant peptides are usually obtained from enzymatic hydrolysates of proteins, with their activities influenced by the species from which the protein is isolated, and the sequence of specific amino acids (Wang et al., 2014). To date, numerous antioxidant peptides from different sources have been isolated and identified. Oysters are economically important shellfish that contain various bioactive polypeptides showing antioxidant (Umayaparvathi et al., 2015), antimicrobial (Seo et al., 2017), immune enhancing (Chen et al., 2013), anti‐angiotensin‐converting enzyme (Cheong et al., 2017; Ug et al., 2018), antitumor (Cheong et al., 2017), and antiproliferative (Aldairi et al., 2018) activities. Antioxidative peptides are mainly concentrated in the species *Crassostrea hongkongensis* (Peng et al., 2020), *Crassostrea gigas* (Qian et al., 2008), *Crassostrea madrasensis* (Asha et al., 2016), *Saccostrea cucullata* (Umayaparvathi et al., 2015), and *Crassostrea talienwhanensis* (Wang et al., 2014). However, no antioxidant peptides have been extracted from *Crassostrea rivularis* yet. The production of *Crassostrea rivularis* in Guangdong, Guangxi, and Fujian provinces has exceeded one million tons in recent years. However, products of *Crassostrea rivular* are mostly entered into market after simple processing except for direct consumption. The economic value has not been fully exploited. The study of bioactive substances is the leading direction of marine research. Natural marine active peptides have high stability. In this study, we used *C. rivularis* as a source to separate antioxidant peptides from its enzymatic hydrolysates and investigated their sequence and antioxidant properties. After enzymatic hydrolysis, we used membrane‐separation and semipreparative reversed‐phase liquid chromatography (semiprep RPLC) to separate functional polypeptides, followed by investigation of their identity, molecular weight (MW) distribution, amino acid composition, and sequence using high‐performance liquid chromatography (HPLC) and ultra‐performance liquid chromatography‐quadrupole/time‐of‐flight tandem mass spectrometry (UPLC Q‐TOF MS/MS). Additionally, we evaluated the antioxidant activities of the peptides and their protective effects against H_2_O_2_‐induced oxidative damage in Caco‐2 cells. The relationship between the sequence and antioxidant activity of the peptides may provide a theoretical basis for the effective preparation of active polypeptides from *C. rivularis*.

## 2 MATERIALS AND METHODS

### 2.1 Materials and reagents

Oysters (*C. rivularis*) were purchased from an aquatic product market in Taishan, Guangdong, and were identified visually as per conventional standards. Sephadex G‐25, 2, 2‐diphenyl‐1‐picrylhydrazyl (DPPH), BHT, ascorbic acid (V_C_), reduced glutathione (GSH), L‐oxidized GSH, bacitracin, [Glu1]‐fibrinopeptide B human, magainin II, aprotinin, cytochrome C, and the cell line Caco‐2 were purchased from Sigma‐Aldrich. Alcalase (210 AU/mg) and trypsase (≥250 U/mg)) were purchased from Novozymes. Microfiltration (MF) membranes and ultrafiltration (UF) membranes were obtained from Fujian Filter and Membrane Technology. Fetal bovine serum (FBS), nonessential amino acids, streptomycin, penicillin, and trypsin–EDTA were purchased from Solarbio Life Sciences. Other reagents used in this study were of analytical or HPLC grade.

### 2.2 Preparation of oyster hydrolysate by enzymatic hydrolysis

Oyster hydrolysates were prepared using an enzymatic method (Yang et al., 2019; Zheng et al., 2016). The oyster homogenates were added to deionized water at a ratio of 1:3.65 (w/v; g:ml) and incubated for 0.5 h. After adjusting the pH to 8.0, homogenate samples were hydrolyzed by alcalase (0.58%) and trypsase (0.22%) for 2 h at 55°C. The reaction mixture was then boiled for 20 min and centrifuged at 10,278 × *g* for 15 min. The supernatant was fractionated sequentially through three MW cut‐off membranes (MWCOM), including one MF membrane (0.22 μm) and two UF membranes (200 kDa and 8 kDa, in that order). The yield of the fractions through the 8 kDa MWCOM was 31.41% ± 0.47%, and the percentage of peptides was 84.03% ± 0.85%, tested by trichloroacetic acid precipitation method (Dingess et al., 2019). The 0–8 kDa fraction was named CRRS‐A, was lyophilized by freeze‐drying, and stored at −20°C before use.

### 2.3 CRRS‐A purification

#### 2.3.1 Gel filtration analysis

The components of CRRS‐A were isolated as described previously (Hu et al., 2021) with some modification. One gram of lyophilized hydrolysate, CRRS‐A, was dissolved in 20 ml water and was isolated using a Sephadex G‐25 gel filtration chromatography column (10 × 400 mm). Two milliliters of the solution was then eluted with Milli‐Q water at a flow rate of 2 ml/min, and the absorbance of the eluent was recorded at 220 nm using an ultraviolet (UV) detector (APD‐M20A; Shimadzu).

#### 2.3.2 RP‐HPLC analysis

The components of CRRS‐A were analyzed according to the previous study (Seo et al., 2013) with some modification. CRRS‐A (20 μl) was fractionated by RP‐HPLC on a Zorbax SB C‐18 column (4.6 × 250 mm; Agilent Technologies,). Elution was performed with a linear gradient of solvent A (0.1% trifluoroacetic acid in Milli‐Q water) to solvent B (0.1% trifluoroacetic acid in methanol), at a flow rate of 1 ml/min, according to the following procedure: 0–5 min, 99% A; 5–10 min, 90% A; 10–16 min, 70% A; 16–27 min, 50% A; 27–37 min, 10% A; and 37–47 min, 99% A. Absorbance was detected at 220 nm by a UV detector (APD‐M20A; Shimadzu).

#### 2.3.3 Semiprep RPLC preparation

The purification of CRRS‐A was prepared with modifications as described previously (Wang et al., 2014). Antioxidant peptides from CRRS‐A (200 mg/ml) were prepared by semiprep RPLC on a semipreparative Zorbax SB C‐18 column (9.4 × 250 mm; Agilent Technologies). Elution was performed with solvent A (Milli‐Q water) and solvent B (methanol) at a flow rate of 4.7 ml/min, and absorbance was detected at 220 nm using a UV detector (PAD 2998; Waters Corp.,). The elution procedure was as follows: 0–5 min, 98% A; 5–10 min, 90% A; 10–15 min, 70% A; 15–20 min, 50% A; 20–30 min, 20% A; and 30–35 min, 98% A. The fractions were dried with a rotary evaporator to remove methanol, followed by lyophilization and storage at −20°C before use.

### 2.4 Determination of MW and amino acid composition

#### 2.4.1 MW

The MW distributions of the fractions were determined using an HPLC system (LC‐20 AD; Shimadzu) with a UV detector (APD‐M20A; Shimadzu) as described previously (Zhu et al., 2017). MW was evaluated on a TSK‐GEL G2000SWXL column (7.8 × 300 mm, 5 μm; Tosoh,). Fractions (0.25 mg/ml) were evaluated with a constant ratio of solvent A (0.1% trifluoroacetic acid in Milli‐Q water) and solvent B (0.1% trifluoroacetic acid in acetonitrile) at 80:20 and a flow velocity of 0.5 ml/min, with the response value detected at 220 nm. The MW standards included reduced GSH (307.3 Da), L‐oxidized GSH (612.6 Da), bacitracin (1422.7 Da), [Glu] fibrinopeptide B human (1570.5 Da), magainin II (2466.9 Da), aprotinin (6511.8 Da), and cytochrome C (12,500.0 Da). The equation for the relation between retention time (t) and MW is given in Equation (1):

(1) $$\mathrm{lg}\left(\mathrm{MW}\right)=-0.0037t^{2}–0.0821t+5.6697,R^{2}=0.9912$$

The peak‐area normalization method was used to determine the MW distribution of the fractions.

#### 2.4.2 Amino acid composition

The method was slightly modified according to the previous study (Carrasco‐Castilla et al., 2012). The fractions (per 200 mg) were hydrolyzed in 6 M HCl containing 0.1% phenol and incubated at 110°C in a sealed container for 22 h. After cooling, the solutions were dried by nitrogen flushing and dissolved in 1 ml 0.01 M HCl. The fractions (per 200 mg) were hydrolyzed in 5 M NaOH and incubated at 110°C in a sealed container for 22 h. After cooling, distilled water was added to the solutions to a final volume of 10 ml. Two milliliters of these solutions was then adjusted to pH 7.0 using 2.5 M HCl, followed by addition of double‐distilled water to a final volume of 5 ml.

The amino acid composition was analyzed on a Zorbax Eclipse AAA column (4.6 × 150 mm, 3.5 μm; Agilent Technologies) using an HPLC system (1100; Agilent Technologies). Samples (5 μl) were analyzed using solvent A (90 mM phosphate buffer solution; pH 7.8) to solvent B (acetonitrile:methanol:Milli‐Q water = 450:450:100) at a flow rate of 2 ml/min, with UV absorption determined at 318 nm (G1315B; Agilent Technologies), and fluorescence absorption determined at an excitation wavelength of 266 nm and an emission wavelength of 305 nm (G1321A; Agilent Technologies).

### 2.5 Structural characterization of purified antioxidative peptides

The active fraction was further purified by UPLC (1290; Agilent Technologies) on an SB‐C18 RRHD column (50 × 2.1 mm, 1.8 μm; Agilent Technologies) as described previously (Yang et al., 2020). Elution was performed using solvent A (Milli‐Q water) and solvent B (methanol) according to the following procedure: 0–1 min, 15% B; 1–4 min, 90% B; 4–10 min, 90% B; and 10–12 min, 15% B. The gradient elution was performed at a flow velocity of 0.2 ml/min, and data were acquired at a wavelength of 220 nm using a UV detector (G7117B; Agilent Technologies).

An accurate amino acid sequence for the purified peptides was determined using a Q‐TOF MS/MS system (maXis Impact; Bruker,) equipped with an electrospray ionization source in positive mode. The molecular mass was determined by a single charged [M + H]^+^ state in the mass spectrum. Spectra were recorded over the mass/charge (m/z) range of 50–2000. The capillary voltage was 3500 V, end‐plate offset was −500 V, charging voltage was 2000 V, the nebulizer was 0.3 bar, the dry heater was set at 180°C, and the flow rate of dry gas was 4.0 L/min. The peptides were fragmented by low‐energy collision‐induced separation to detect peptide fragments for de novo sequencing.

### 2.6 Antioxidant assays

#### 2.6.1 Determination of reducing power

The reducing power was measured as described by Umayaparvathi, Meenakshi, Vimalraj, Arumugam, Sivagami, and Balasubramanian (2014). Briefly, 1 ml of the solution was mixed with 2.0 ml phosphate buffer (0.2 M; pH 6.6) and 1 ml potassium ferricyanide solution (1%), followed by vortexing for 1 min and incubation at 50°C for 20 min. After incubation, 1 ml trichloroacetic acid (1%) was added and centrifuged at 13,360 × *g* for 10 min, after which, 1 ml of the supernatant was mixed with 1 ml distilled water and 0.2 ml ferric chloride (0.1%) and incubated at 50°C for 10 min. The response value of the samples was read at optical density (OD)700 nm using an ELISA reader (Sunrise‐basic; Tecan, Männedorf,). Increased absorbance indicated enhanced reducing power. BHT, GSH, and V_C_ were used as positive controls.

#### 2.6.2 DPPH radical scavenging activity

DPPH radical‐scavenging activity was estimated as described by Mirzaei et al. (2015) and Khan et al. (2018). Samples (2 ml) were added to 2.0 ml of 0.1 mM DPPH solution (sample group) or 2.0 ml ethanol (control group), and 2.0 ml ethanol was added to 2.0 ml DPPH solution for the blank group. The mixtures were then vortexed for 1 min and incubated at 37°C for 60 min in the dark, after which the absorbance was read at 517 nm (Sunrise‐basic; Tecan). Lower optical density indicated higher radical‐scavenging activity. The ability to scavenge the DPPH radical was calculated using the following equation:

(2) $$K_{D}=\frac{A_{B}-\left(A_{S}-A_{C}\right)}{A_{B}}\times 100,$$

where *K*_*D*_ is the rate of DPPH radical‐scavenging activity (%), *A*_*B*_ is the OD of the blank group, *A*_*S*_ is the OD of the sample group, and *A*_*C*_ is the OD of the control group. BHT, GSH, and V_C_ served as positive controls.

#### 2.6.3 Hydroxyl radical scavenging activity

The effect of hydroxyl radicals was measured using the 2‐deoxyribose oxidation method (Zhang et al., 2017). The reaction mixture contained 0.2 ml of 0.15 M sodium phosphate buffer (pH 7.4), 0.3 ml of 5 mM phenanthroline dissolved in ethanol, 0.3 ml of 0.75 mM FeSO_4_, 0.20 ml of 10 mM hydrogen peroxide, 1 ml of distilled water, and 1 ml of sample solutions in a tube. The reaction was started by adding hydrogen peroxide. The reaction solution was incubated at 37°C for 1 min and the reaction was stopped by adding 0.2 ml of 0.1% hydrogen peroxide. As a blank control, the 0.3 ml distilled water was replaced with 0.3 ml phenanthroline dissolved in ethanol, 0.3 ml distilled water replaced with FeSO_4_, and 0.2 ml distilled water replaced with 0.2 ml hydrogen peroxide. As another control, 0.2 ml distilled water was replaced with 0.2 ml hydrogen peroxide. For the control tube, 1 ml distilled water was replaced with 1 ml sample solution. Sample absorbances were measured at 550 nm (Sunrise‐basic Tecan,). The percentage of inhibition was computed using the following equation:

(3) $$K_{H}=\frac{A_{C}-A_{S}}{A_{N}-A_{B}}\times C_{S}\times \frac{1}{V}\times D,$$

where *K*_*H*_ is the rate of hydroxyl radical‐scavenging activity (U/ml), *A*_*C*_ is the OD of the control group (distilled water replaced H_2_O_2_), *A*_*S*_ is the OD of the test sample (added sample), *A*_*N*_ is the OD of the reaction without sample (distilled water used as the sample), *A*_*B*_ is the OD of the buffer and distilled water, *C*_*S*_ is the concentration of the H_2_O_2_ solution, *V* is the volume of the sample used, and *D* is the fold‐dilution of the sample. BHT, GSH, and V_C_ served as positive controls.

#### 2.6.4 Superoxide radical scavenging activity

The rate of inhibition of superoxide radicals was determined using an anti‐superoxide anion kit (Nanjing Jiancheng Bioengineering Institute,). The percentage of inhibition was calculated using the following equation:

(4) $$K_{S}=\frac{A_{C}-A_{S}}{A_{C}-A_{T}}\times C_{S}\times 1000\times D,$$

where *K*_*S*_ is the rate of superoxide radical‐scavenging activity (U/g), *A*_*C*_ is the OD of the control group (distilled water), *A*_*S*_ is the OD of the test sample group, *A*_*T*_ is the OD of the standard (vitamin C liquor), *C*_*S*_ is the concentration of the vitamin C liquor, and *D* is the concentration of the sample. BHT, GSH, and V_C_ served as positive controls.

### 2.7 Protective effects of F5 on Caco‐2 cells damaged by H_2_O_2_

Experiments were performed using Caco‐2 (human colon cancer) cell lines (Sigma‐Aldrich). Caco‐2 cells were grown as a monolayer in minimum essential medium with Earle’s balanced salt solution containing 10% FBS, 1% penicillin and streptomycin, and 1% nonessential amino acid at 37°C in a 5% CO_2_ atmosphere. Complete medium was replaced every 2 days before collecting the cells with 0.25% trypsin EDTA.

Cell viability of the damaged Caco‐2 cells treated with F5 and those treated with the control group was tested by 3‐(4, 5‐dimethyl thiazol‐2‐yl)‐2,5‐diphenyltetrazolium bromide (MTT) assay (Wang et al., 2012). The experiment involved first incubating F5 and complete medium for 4 h, and then using 10 mM H_2_O_2_ to damage Caco‐2 cells for 6 h on 96‐well round‐bottom microplates (Wang et al., 2015). The control group (without F5 or H_2_O_2_) was incubated with complete medium for 10 h. The microplates were put in a 5% CO_2_ cell incubator at 37°C for 4 h after adding 20 μl 5 mg/ml MTT solution. After that, the supernatant was slowly removed with pipet and the plates were washed twice with PBS; the cells were lysed by adding 200 μl DMSO to each well. The microplates were then shaken on a microplate oscillator for 15 min. The OD was recorded at a wavelength of 570 nm (Sunrise‐basic; Tecan), and the percentage of cell viability was calculated using the following equation:

(5) $$K_{V}=\frac{A_{C}-A_{B}}{A_{S}-A_{B}}\times 100,$$

where *K*_*V*_ is the rate of cell viability (%); *A*_*C*_ is the OD of the cells, MTT, and sample; *A*_*B*_ is the OD of MTT and medium without cells; and *A*_*S*_ is the OD of cells and MTT without sample.

### 2.8 Statistical analysis

All experiments were performed in triplicate, and data are expressed as mean ± standard deviation. Differences among treatments were analyzed by one‐way analysis of variance with a multiple‐comparison Tukey test using SPSS software (v.18.0; SPSS Inc.,).

## 3 RESULTS

### 3.1 Purification of peptides from CRRS‐A and their MW distributions

**FIGURE 1:**
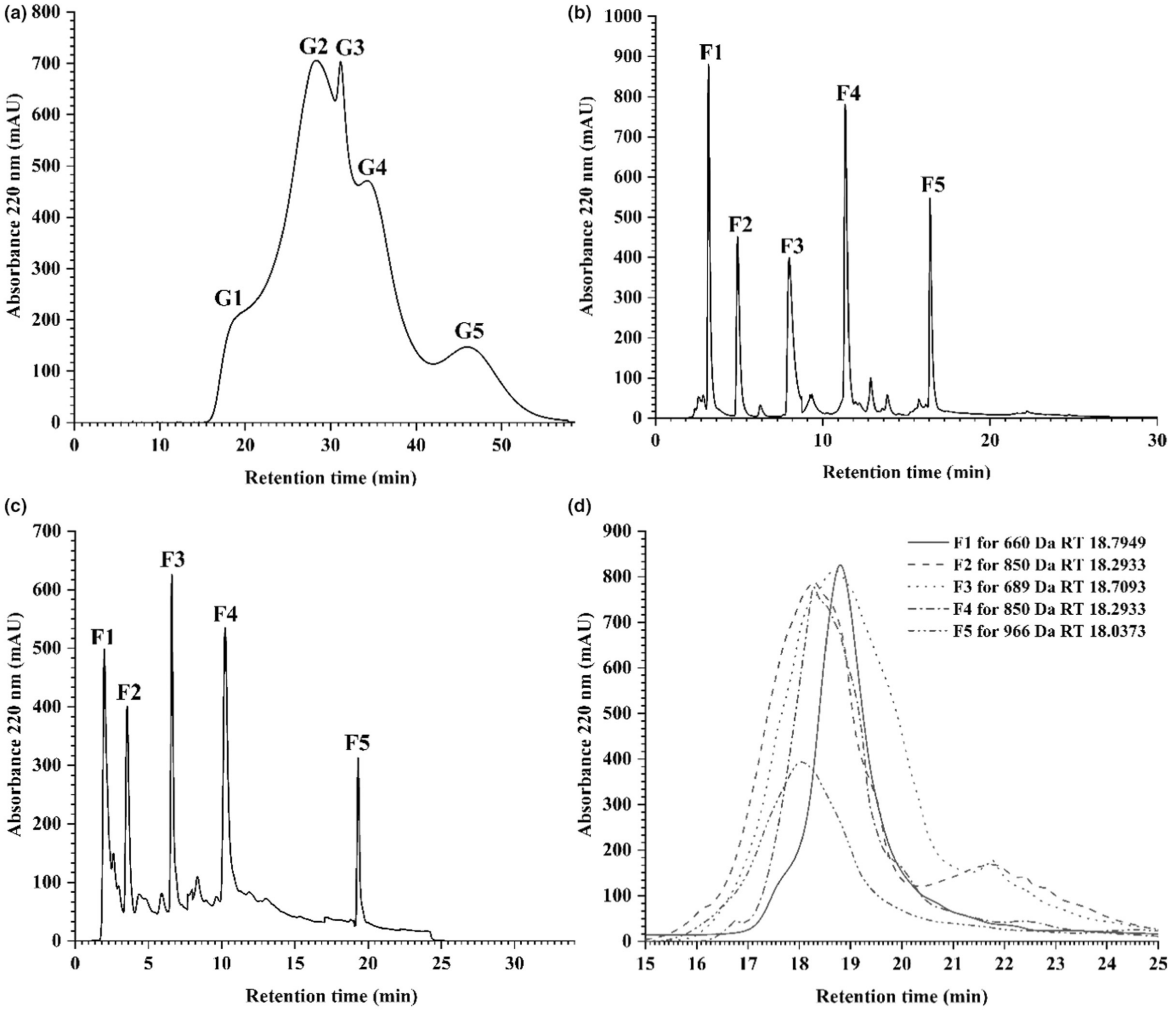
Chromatography of CRRS‐A separated by Sephadex G‐25 column (a), reversed‐phase high‐performance liquid chromatography (b) and semiprep reversed‐phase liquid chromatography (c). Molecular weight distributions of five fractions collected by semiprep reversed‐phase liquid chromatography (d)

The 0–8 kDa ultrafiltration group CRRS‐A was separated by Sephadex G‐25 and RP‐HPLC, and different CRRS‐A components were separated by MW (G1, G2, G3, G4, and G5; Figure 1a). Because these components could not be clearly separated, RP‐HPLC was used to separate them according to polarity, resulting in five fractions (F1, F2, F3, F4, and F5; Figure 1b). These fractions were then collected by semiprep RPLC (Figure 1c). The MW distributions for F1 through F5 are shown in Figure 1d. Each fraction showed a relatively concentrated molecular weight distribution (F1, F2, F3, F4, and F5 peaks: 660, 850, 689, 850, and 966 Da, respectively).

### 3.2 Amino acid composition of F1 through F5

The amino acid compositions of F1 through F5 from the hydrolysis mixture of *C. rivularis* are shown in Table 1. CRRS‐A contained both essential and nonessential amino acids, with abundant hydrophobic amino acids, including proline (P), leucine (L), phenylalanine (F), methionine (M), valine (V), tyrosine (Y), and alanine (A), and hydrophilic amino acids, such as serine (S), cysteine (C), glycine (G), and threonine (T). Interestingly, F4 mainly comprised P, with a relative content of 96.11%, suggesting potential use of F4 as a dietary supplement with P.

**TABLE 1:** Amino acid composition of F1‐F5 from the hydrolysate of *Crassostrea rivularis*

Amino acid	F1	F2	F3	F4	F5	CRRS‐A
	Relative content %					
Aspartic acid (D)	1.88 ± 0.44	4.62 ± 0.09	5.56 ± 0.34	0.18 ± 0.09	2.77 ± 0.64	4.19 ± 0.56
Glutamic acid (E)	6.57 ± 1.35	10.47 ± 0.19	7.39 ± 0.39	0.33 ± 0.15	5.55 ± 1.13	4.92 ± 0.83
Serine (S)	3.13 ± 0.67	3.59 ± 0.07	5.69 ± 0.32	0.13 ± 0.06	4.52 ± 0.96	3.17 ± 0.18
Histidine (H)	10.74 ± 2.01	0.34 ± 0.01	13.48 ± 1.01	0.03 ± 0.03	16.02 ± 2.96	7.81 ± 0.91
Glycine (G)	2.05 ± 0.59	14.32 ± 0.24	3.47 ± 0.21	0.47 ± 0.19	2.36 ± 0.67	2.55 ± 0.38
Threonine (T)^a^	1.94 ± 0.45	2.71 ± 0.07	6.70 ± 0.41	0.15 ± 0.09	2.00 ± 0.46	2.79 ± 0.64
Arginine (R)	1.74 ± 0.37	3.40 ± 0.06	1.45 ± 0.08	0.20 ± 0.09	3.26 ± 0.68	3.93 ± 0.66
Alanine (A)^b^	11.89 ± 2.35	6.17 ± 0.11	0.41 ± 0.02	0.20 ± 0.08	12.86 ± 2.51	1.20 ± 0.03
Tyrosine (Y)^b^	2.69 ± 0.67	3.78 ± 0.08	2.21 ± 0.30	0.13 ± 0.07	3.50 ± 0.86	2.40 ± 0.47
Cysteine (C)	4.15 ± 1.59	1.15 ± 0.02	4.05 ± 0.24	0.04 ± 0.02	0.07 ± 0.02	0.54 ± 0.16
Valine (V)^a^ ^,^ ^b^	1.93 ± 0.32	3.15 ± 0.04	17.56 ± 0.77	0.26 ± 0.09	5.24 ± 0.88	3.76 ± 0.12
Methionine (M)^a^ ^,^ ^b^	0.10 ± 0.23	0.50 ± 0.01	2.21 ± 0.13	0.02 ± 0.01	1.91 ± 0.45	0.77 ± 0.38
Tryptophan (W)^a^ ^,^ ^b^	0.42 ± 0.11	–	0.39 ± 0.02	0.49 ± 0.25	0.12 ± 0.02	0.95 ± 0.19
Phenylalanine (F)^b^	3.00 ± 0.65	2.48 ± 0.04	–	0.26 ± 0.12	3.05 ± 0.65	2.03 ± 0.35
Isoleucine (I)^a^ ^,^ ^b^	0.04 ± 0.02	1.18 ± 0.02	1.40 ± 0.09	0.07 ± 0.03	0.61 ± 0.14	1.30 ± 0.48
Leucine (L)^a^ ^,^ ^b^	5.90 ± 1.26	11.75 ± 0.23	–	0.81 ± 0.38	8.04 ± 1.70	3.03 ± 0.51
Lysine (K)^a^	8.83 ± 1.56	5.92 ± 0.09	–	0.10 ± 0.03	12.44 ± 2.18	4.05 ± 0.67
Proline (P)^a^ ^,^ ^b^	33.00 ± 0.55	24.48 ± 0.03	28.03 ± 0.12	96.11 ± 3.58	15.68 ± 2.59	50.61 ± 0.69

^a^ Essential amino acids.

^b^ Hydrophobic amino acid; − not detected.

### 3.3 Sequence determination of F1 through F5

**FIGURE 2:**
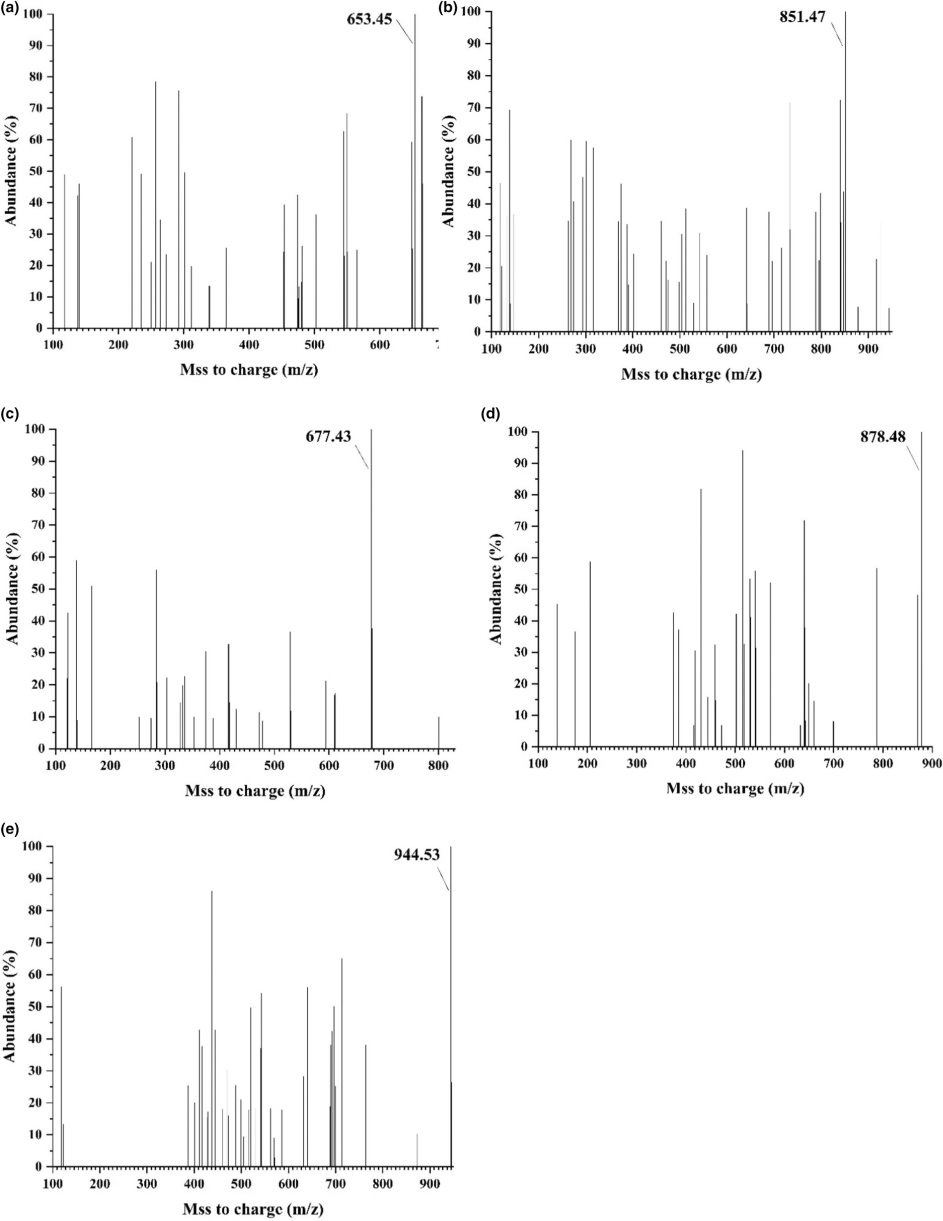
MS spectrum of F1 (a), F2 (b), F3 (c), F4 (d), and F5 (e) from the hydrolysate of *Crassostrea rivularis*

**FIGURE 3:**
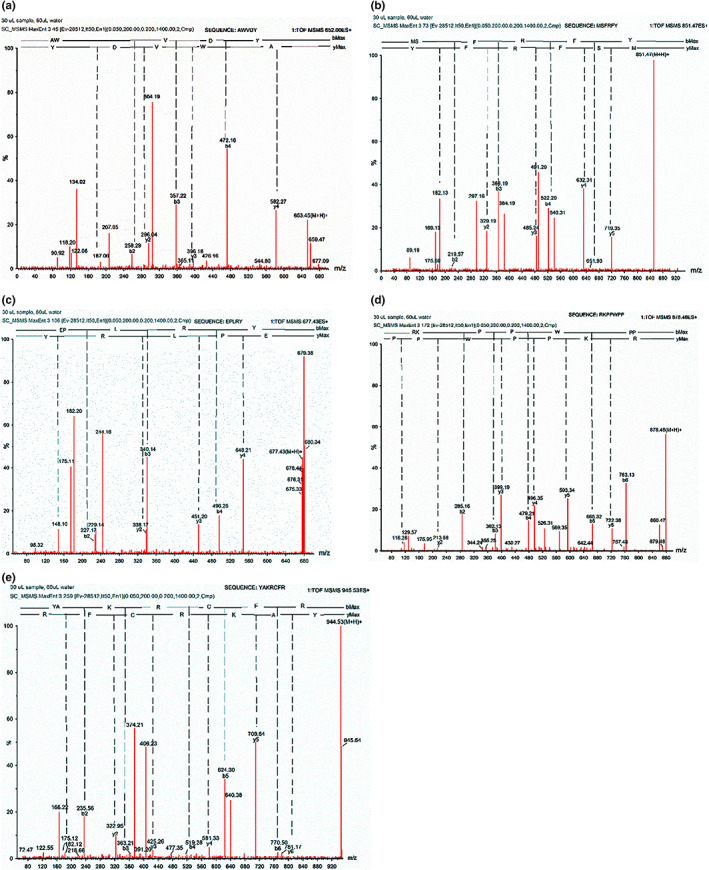
Identification of molecular mass and amino acid sequence of F1 (a), F2 (b), F3 (c), F4 (d), and F5 (e) from the hydrolysate of *Crassostrea rivularis*

Acquisition of the MS spectra for F1 through F5 revealed the highest‐abundance ion peaks at 653.45, 851.47, 677.43, 878.48, and 944.53 Da, respectively (Figure 2), which were chosen as the parent ion peaks in the secondary mass spectrum. A major single charge ion ([M + H]^+^) with m/z values of 653.45, 851.47, 677.43, 878.48, and 944.53 was observed in each MS spectrum. The results of identification with bMax ions and yMax ions are shown in Figure 3. Amino acid sequences for F1 through F5 determined from the MS/MS spectra identified the highest‐abundance peptides of F1 through F5 as having specific molecular masses of 652 Da, 850 Da, 676 Da, 877 Da, and 943 Da, respectively. F1 contained the MS/MS irons of y4 (M‐A + H_2_O, m/z = 582.27 Da), y3 (M‐AW+H_2_O, m/z = 396.18), y2 (M‐AWV + H_2_O, m/z = 296.04 Da); F2 contained the y5 iron (M‐M + H_2_O, m/z = 719.35 Da), y4 (M‐MS + H_2_O, m/z = 632.31 Da), y3 (M‐MSF + H_2_O, m/z = 485.24 Da), y2 (M‐MSFR+H_2_O, m/z = 329.18 Da); F3 contained the y4 iron (M‐E + H_2_O, m/z = 548.21 Da), y3 (M‐EP + H_2_O, m/z = 451.20 Da), y2 (M‐EPL + H_2_O, m/z = 338.17 Da); the same y iron type could be seen in F4: y6 (M‐R + H_2_O, m/z = 722.38 Da), y5 (M‐RK + H_2_O, m/z = 593.34 Da), y4 (M‐RKP + H_2_O, m/z = 496.35 Da), y3 (M‐RKPP+H_2_O, m/z = 399.19 Da), y2 (M‐RKPPW+H_2_O, m/z = 213.58 Da); the y irons were seen in F5 the same as F5, y6 (M‐Y + H_2_O, m/z = 770.50 Da), y5 (M‐YA + H_2_O, m/z = 709.54 Da), y4 (M‐YAK+H_2_O, m/z = 581.33 Da), y3 (M‐YAKR+H_2_O, m/z = 425.26 Da), y2 (M‐YAKRC+H_2_O, m/z = 322.95 Da). According to the results, the sequences of these peptides were confirmed as AWVDY(F1), MSFRFY(F2), EPLRY(F3), RKPPWPP(F4), and YAKRCFR(F5). Hydrophobic amino acids alanine (A), tryptophan (W), valine (V), tyrosine (Y), methionine (M), phenylalanine (F), and leucine (L) were identified from the sequences of F1 through F5.

### 3.4 Antioxidant activities of F1 through F5 and the protective effects of F5 in damaged Caco‐2 cells

**FIGURE 4:**
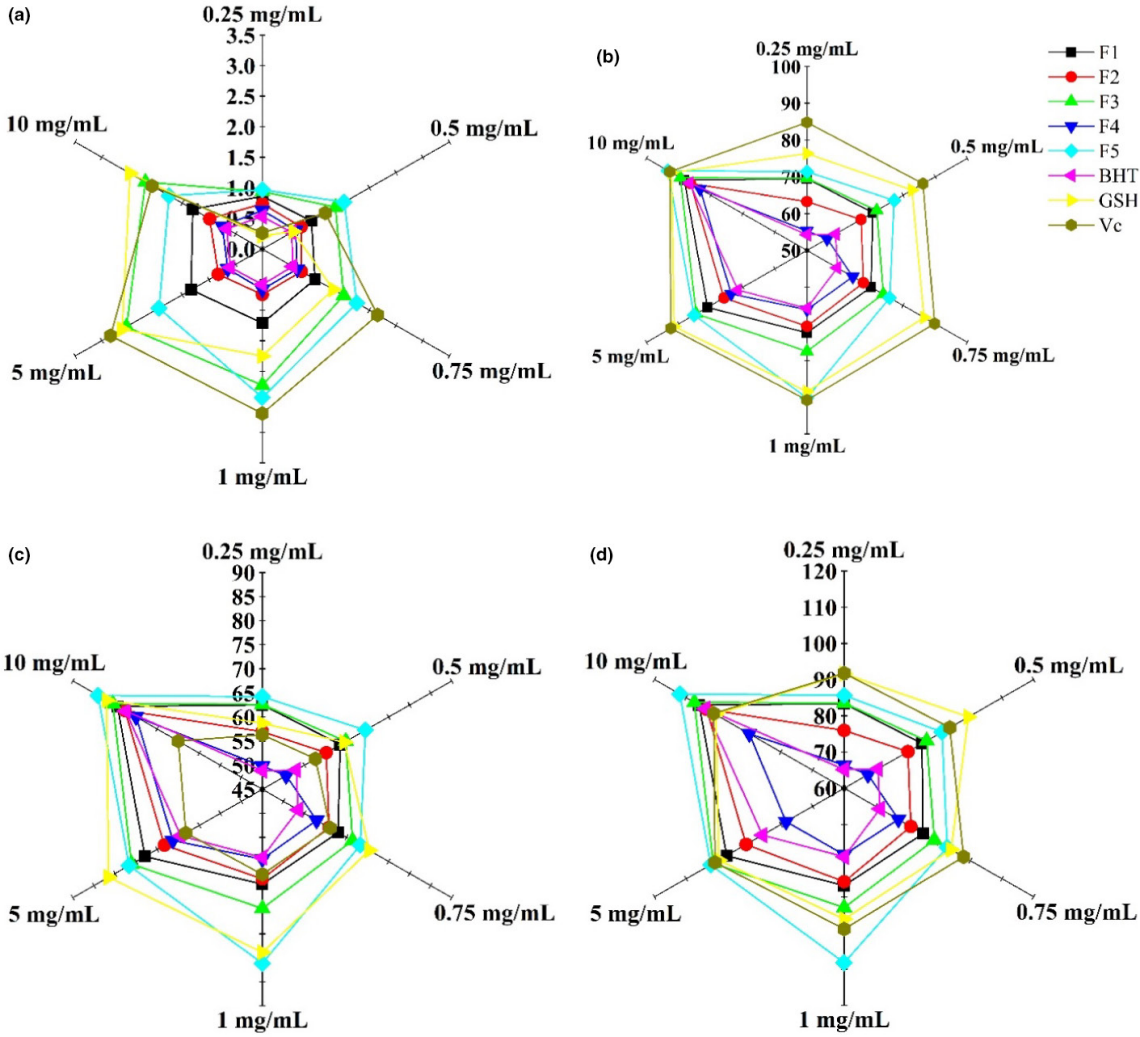
Reducing power (a), DPPH radical scavenging activity (b), hydroxyl radical scavenging activity (c), and superoxide radical scavenging activity (d) of fractions collected (F1–F5) from the hydrolysate of *Crassostrea rivularis* and positive control groups (BHT, GSH, and V_C_)

Figure 4a shows the reducing power of F1 through F5 and the positive control groups (BHT, GSH, and V_C_), with increases in absorbance indicating greater reducing power. Different concentrations of F1 through F5 showed various reducing capacities in a dose‐dependent manner. F5 showed the highest reducing power at 1 mg/ml, relative to the other peptides and the positive control groups (GSH and BHT); however, at concentrations ≥5 mg/ml, F3 showed the highest reducing power with an OD_700_ of 2.54 (5 mg/ml). DPPH is a stabilized free radical and takes over an electron or hydrogen radical to become a stable intact molecule (Lavanya & Ganapathy, 2019). Figure 4b shows the DPPH‐scavenging activities of F1 through F5 and the positive controls (BHT, GSH, and V_C_). As the concentrations increased, DPPH radical‐scavenging activity significantly increased, with F5 showing the highest scavenging activity relative to the other peptides and BHT at various concentrations. Additionally, F5 showed higher DPPH radical‐scavenging activity than GSH but lower than V_C_ at 1 mg/ml, whereas F5 showed the highest DPPH radical‐scavenging activity among all other peptides and all positive controls at 10 mg/ml (93.36%). Hydroxyl radical is a type of ROS produced in a Fenton reaction and capable of injuring several cellular constituents (Je et al., 2007). Figure 4c shows the hydroxyl radical‐scavenging activities of F1 through F5 and the positive controls (BHT, GSH, and V_C_), revealing higher activities for F1, F2, F3, and F5 than those of BHT (*p* < .001) and V_C_ (*p* < .05) at the same concentrations. Moreover, F5 exhibited similarly high hydroxyl radical‐scavenging activity as GSH, with the highest activity observed in F5 at 10 mg/ml (84.02 U/ml). Similarly, superoxide radical‐scavenging activity increased in a dose‐dependent manner (Figure 4d), with F1, F2, F3, and F5 showing better superoxide radical‐scavenging activity than BHT and F5 showing better activity than the other peptides and all positive controls at 1 mg/ml, 5 mg/ml, and 10 mg/ml (highest activity: 112.03 U/g at 10 mg/ml).

The 50% inhibitory concentration (IC_50_) values of F1 through F5 for eliminating various free radicals are shown in Table 2. The hydrolysate obtained by enzymolysis of *C. rivularis* indicated high antioxidant activity similar to that of BHT, GSH, and V_C_, and there was no significant difference in the IC_50_ values of DPPH between F5, GSH, and V_C_. Similar to V_C_, F5 possessed better hydroxyl radical‐scavenging activity than the other fractions, with the IC_50_ of hydroxyl radical reaching 18.75 μg/ml. Moreover, there was no significant difference among F5, F2, F3, F1, and GSH in the IC_50_ of superoxide radicals, with all achieving satisfactory effects at scavenging superoxide radicals, and F5 showing the highest activity.

**TABLE 2:** IC_50_ values of various free radical of F1–F5 from the hydrolysate of *Crassostrea rivularis*

	IC_50_ (μg/ml)		
	DPPH	Hydroxyl	Superoxide
F1	27.50 ± 4.55^bc^	44.00 ± 6.04^bc^	18.00 ± 3.08^a^
F2	50.75 ± 7.12^c^	89.75 ± 22.85^d^	16.5 ± 1.52^a^
F3	28.25 ± 5.35^bc^	36.50 ± 7.79^abc^	16.5 ± 3.84^a^
F4	166.75 ± 8.52^d^	303.75 ± 16.06^e^	84.00 ± 5.42^c^
F5	21.75 ± 7.08^ab^	18.75 ± 6.49^ab^	11.00 ± 2.65^a^
BHT	201.00 ± 10.41^e^	350.75 ± 9.09^f^	56.25 ± 7.22^bc^
GSH	7.50 ± 4.97^ab^	60.75 ± 9.41^cd^	6.50 ± 7.88^a^
V_C_	0.75 ± 0.43^a^	11.25 ± 5.97^a^	35.25 ± 3.34^ab^

*Note*: Values with *p* < .05 were considered significant; values with *p* < .01 were considered extremely significant.

Different lowercase letters in the same column indicate significant differences (*p* < .05).

**FIGURE 5:**
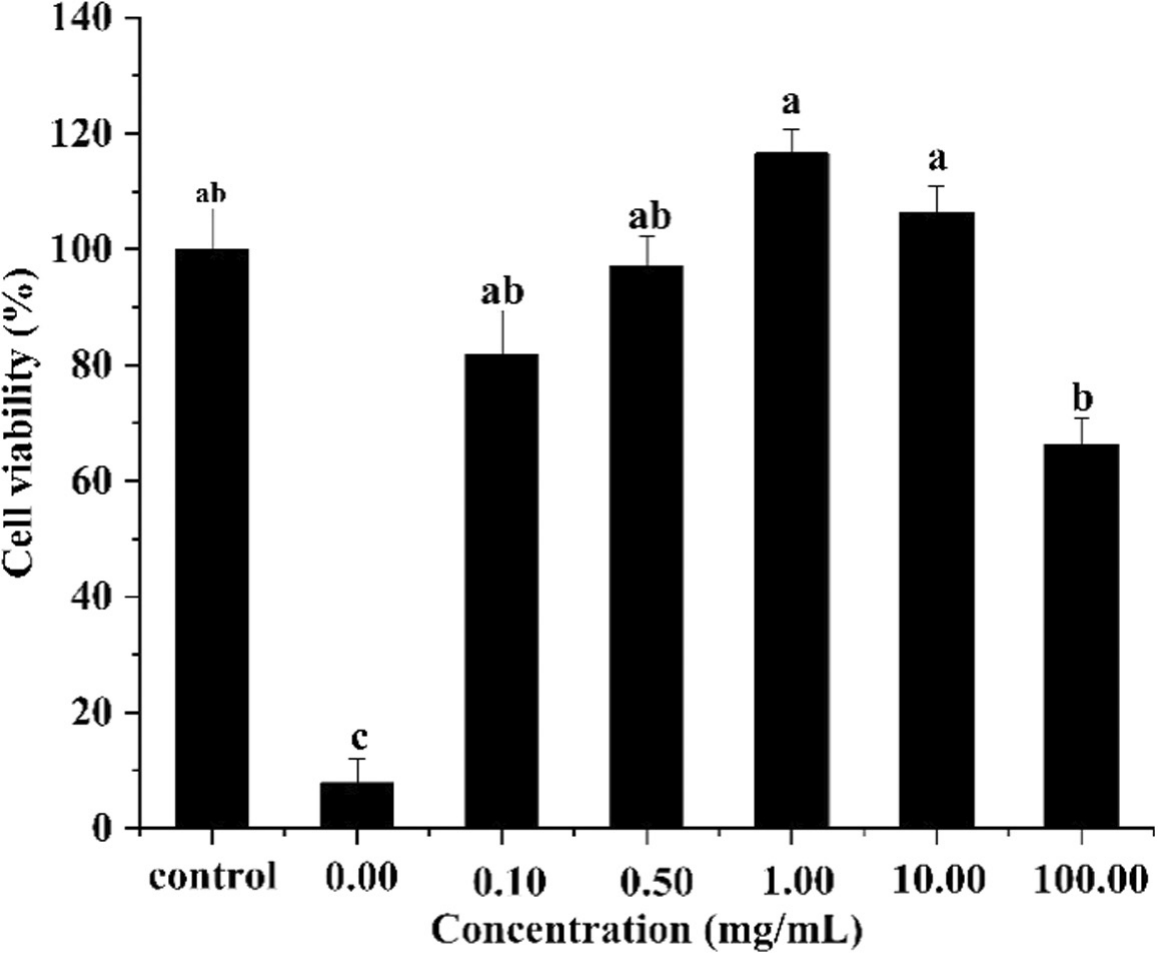
Effect of F5 from the hydrolysate of *Crassostrea rivularis* on the cell viability of H_2_O_2_‐induced Caco‐2 cells

Given these findings, we chose F5 to determine its ability to protect Caco‐2 cells from H_2_O_2_‐induced oxidative damage (Figure 5). Increasing F5 concentrations initially resulted in increased cell viabilities, followed by subsequent decreases, with the highest cell viability observed at 1.0 mg/ml F5. Compared with the control group (0 mg/ml of F5), we found that 0.10 mg/ml–100.0 mg/ml F5 protected H_2_O_2_‐damaged Caco‐2 cells by maintaining their viability, with optimal results observed at a range of 0.50 mg/ml–10.00 mg/ml (cell viabilities >90%). Interestingly, compared with the control group, 1.00 mg/ml and 10.00 mg/ml F5 not only prevented cell injury, but also promoted cell growth.

## 4 DISCUSSION

Oysters are generally used as a source to prepare antioxidative peptides. In this study, five antioxidative peptides (F1–F5) were obtained from CRRS‐A of *C. rivularis* by MWCOM and semiprep RPLC (Figure 1). Sequences with the highest‐abundance ion‐peaks [AWVDY (F1), MSFRFY(F2), EPLRY(F3), RKPPWPP(F4), and YAKRCFR(F5)] were identified by UPLC Q‐TOF MS/MS, with MWs of 652 Da, 850 Da, 676 Da, 877 Da, and 943 Da, respectively (Figures 2 and 3), in agreement with the MW distribution of the fractions (Figure 1). The five peptides, especially F5, possessed high antioxidant activities, similar to BHT, GSH, and V_C_ (Figure 4 and Table 2).

Reducing power and DPPH, hydroxyl, and superoxide radical‐scavenging activities are important indexes for measuring antioxidant capacity. Peptides with a higher reducing power have a greater ability to contribute electrons or hydrogen, therefore acting as good antioxidative agents (Je et al., 2009). A previous study reported that peptides from oysters (*S. cucullata*) show good reducing power and reach an OD_700_ of 2.63 (Umayaparvathi, Meenakshi, Vimalraj, Arumugam, & Balasubramanian, 2014). In the present study, we showed similar results, with F3 and F5 demonstrating reducing powers at OD_700_ values of 2.54 and 2.43, respectively. Moreover, F5 possessed high DPPH, hydroxyl, and superoxide radical‐scavenging activities of 90.16%, 81.15 U/ml, and 108.20 U/g at 1 mg/ml, respectively, with similar activities not achieved by the other peptides. In contrast, the DPPH radical‐scavenging activity of the peptide from *S. cucullata* at 1 mg/ml was only 85.7% (Umayaparvathi et al., 2015). Umayaparvathi, Arumugam, Meenakshi, Dräger, Kirschning, and Balasubramanian (2014) identified three antioxidant peptides from oysters with DPPH radical‐scavenging activities of 82.85%, 74.59%, and 75.67%, respectively. Additionally, peptides from scallop skirts exhibit antioxidant activities for DPPH, hydroxyl, and superoxide radicals of 75.6%, 79.5%, and 76.7%, respectively (Wang et al., 2017).

To gain insight into the relationships between antioxidant activity and peptide sequence, we evaluated the amino acid composition and sequences of the five identified peptides. Amino acid composition has an important effect on the antioxidant activity of peptides (Umayaparvathi et al., 2015). Previous reports show that the presence of hydrophobic amino acid residues is essential for antioxidant peptides (Shi et al., 2017; Umayaparvathi, Arumugam, Meenakshi, Dräger, Kirschning, & Balasubramanian, 2014; Wang et al., 2014). In the present study, F1 through F5 were collected by RPLC due to different polarities, with their respective sequences (the highest‐abundance ion peak) identified as AWVDY, MSFRFY, EPLRY, RKPPWPP, and YAKRCFR. All peptides were abundant in hydrophobic amino acids, including Y residues present in the N‐terminus, which were previously reported as being involved in antioxidant activity (Zhang et al., 2017). Moreover, we showed that F5 (YAKRCFR) possessed the highest antioxidant activity. Lysine (K) is a hydrogen donor that reacts with free radicals to maintain cell stability, with Xia et al. (2017) reporting a relationship between increased K levels and higher antioxidant activity. In the present study, amino acid‐composition assays showed that F1, F2, and F5 sequences were rich in K (8.83%, 5.92%, and 12.44%, respectively), with F4 (RKPPWPP) and F5 (YAKRCFR) also harboring these residues. Je et al. (2009) identified the antioxidant peptide VKAGFAWTANQQLS that showed DPPH, hydroxyl, and superoxide radical‐scavenging activities, with the highest activity observed for hydroxyl radical. In the present study, we identified a similar combination of “AW” residues in F1, which exhibited better antioxidant activity than BHT. Furthermore, Hao et al. (2013) reported the abundance of P and histidine (H) in antioxidant peptides. In the present study, we found that F5 contained H and P at 16.02% and 15.68%, respectively, and exhibited the highest antioxidant activity relative to the other identified peptides and the positive controls at 1 mg/ml. Importantly, F5 (0.10–100.00 mg/ml) promoted an increase in the viability of Caco‐2 cells under oxidative stress conditions, suggesting its possible efficacy as an antioxidant food additive to protect against oxidative damage in the intestines.

## 5 CONCLUSIONS

In summary, we isolated five peptides (F1–F5) using 8 kDa MWCOM and semiprep RPLC and characterized their sequence and antioxidant activities. We found that all five peptides possessed high antioxidant activity similar to positive controls. The MW distributions for F1 through F5 are 660, 850, 689, 850, and 966 Da, respectively. The sequences of these peptides were confirmed as AWVDY(F1), MSFRFY(F2), EPLRY(F3), RKPPWPP(F4), and YAKRCFR(F5).F5 exhibiting the highest scavenging activity for DPPH radical (IC_50_ = 21.75 μg/ml), hydroxyl radical (IC_50_ = 18.75 μg/ml), and superoxide radical (IC_50_ = 11.00 μg/ml). Furthermore, F5 not only remarkably prevented cell injury in the presence of oxidative stress but also promoted cell growth, indicating its potential as an antioxidant food additive for relieving oxidative damage caused by processed foods.

## ACKNOWLEDGEMENT

Thanks to the students and staff of the Key Laboratory of Aquatic Product Processing, Ministry of Agriculture and Rural Affairs, South China Sea Fisheries Research Institute of Chinese Academy of Fishery Sciences for their help in the study.

## FUNDING INFORMTION

This work was supported by the Central Public‐interest Scientific Institution Basal Research Fund, CAFS(grant numbers 2020XT0501, 2020TD69, 2020TD73, and 2019ZD1001); Guangdong MEPP Fund (grant number GDOE[2019]A25); the Science and Technology Planning Project of Guangdong Province (grant number 2017B020204001); Natural Science Foundation of Guangdong Province (grant number 2016A030313144); Program of Key Laboratory of Aquatic Product Processing, Ministry of Agriculture and Rural Affairs (grant number NYJG201305); Young and middle‐aged teachers' basic ability improvement project of Guangxi (grant number 2017KY0801); and Guangxi Natural Science Foundation Program (grant number 2018GXNSFBA2940150); Pearl River S & T Nova Program of Guangzhou (grant number 201906010081).

## CONFLICTS OF INTEREST

The authors declare no conflicts of interest for publishing this manuscript.
